# Zoonoses and One Health: A Review of the Literature

**DOI:** 10.1155/2014/874345

**Published:** 2014-01-30

**Authors:** Satesh Bidaisee, Calum N. L. Macpherson

**Affiliations:** ^1^Department of Public Health and Preventive Medicine, St. George's University, School of Medicine, St. George's, Grenada; ^2^Windward Islands Research and Education Foundation, St. George's, Grenada; ^3^Department of Microbiology, St. George's University, School of Medicine, St. George's, Grenada

## Abstract

*Background*. One health is a concept that was officially adopted by international organizations and scholarly bodies in 1984. It is the notion of combining human, animal, and environmental components to address global health challenges that have an ecological interconnectedness. *Methods*. A cross-sectional study of the available literature cited was conducted from January 1984 when the one health concept was adopted till December 2012 to examine the role of the one health approach towards zoonoses. Inclusion criteria included publications, professional presentations, funding allocations, official documentation books, and book chapters, and exclusion criteria included those citations written outside the period of review. *Results*. A total of 737 resources met the inclusion criteria and were considered in this review. Resources showed a continuous upward trend for the years from 2006 to 2012. The predominant resources were journal publications with environmental health as the significant scope focus for one health. There was also an emphasis on the distribution of the work from developed countries. All categories of years, resources, scopes, and country locale differed from the means (*P* = 0.000). Year of initiative, scope, and country locale showed a dependent relationship (*P* = 0.022, *P* = 0.003, and *P* = 0.021, resp.). *Conclusion*. Our findings demonstrate the rapid growth in embracing the concept of one health, particularly in developed countries over the past six years. The advantages and benefits of this approach in tackling zoonoses are manifold, yet they are still not seemingly being embraced in developing countries where zoonoses have the greatest impact.

## 1. Introduction

One health is a concept that aims to bring together human, animal, and environmental health. Researchers including Louis Pasteur and Robert Koch and physicians such as William Osler and Rudolph Virchow demonstrated the collaborative links between animal and human health. More recently, Calvin Schwabe revived the concept of One Medicine [1]. As the traditional boundaries between medical and veterinary practice continue to pervade society there is a need for the practical application of one health.

One health is defined by the One Health Commission [2] as “the collaborative effort of multiple disciplines to obtain optimal health for people, animals, and our environment.” In another definition, the One Health Initiative Task Force (OHITF) [3] defines one health as “the promotion, improvement, and defense for the health and well-being of all species by enhancing cooperation and collaboration between physicians, veterinarians, and other scientific health professionals and by promoting strengths in leadership and management to achieve these goals.”

The one health approach plays a significant role in the prevention and control of zoonoses. It has been noted by the World Health Organization (WHO) [4] and Graham et al. [5] that approximately 75% of new emerging human infectious diseases are defined as zoonotic, meaning that they may be naturally transmitted from vertebrate animals to humans. New and reemerging zoonoses have evolved throughout the last three decades partly as a consequence of the increasing interdependence of humans on animals and their products and our close association with companion animals. Zoonoses should therefore be considered the single most critical risk factor to human health and well-being, with regard to infectious diseases. Of the 1,461 infectious diseases recognized to occur in humans by the National Academy of Sciences, Institute of Medicine [6], approximately 60% are caused by multihost pathogens, characterized by their movement across various species. This gives significant credence to the importance of examining health effects across species, in order to fully understand the public health and economic impact of such diseases and to help implement treatment and preventive programs. The one health concept is a broad term that covers a variety of subcategories identified as bioterrorism, animals as predictors for disease, and the psychological bonds that can exist between an animal and a human [7].

Zoonoses comprised the primary focus for this review with the overall objective to determine the status of the one health approach and its applications to zoonoses, using scholarly peer-reviewed literature that has been published since the global adoption of the concept in 1984 (for study purposes, January 1, 1984, until December 31, 2012). Four subobjectives were considered. The first assessed scholarly resources on the one health approach published works between January 1, 1984, and December 31, 2012. One health scholarly resources were classified as peer-reviewed publications, professional presentations, grants or funding allocations, reports from the WHO, and books or book chapters. The second objective examined the preferred scope of one health published works within the period of study. Scopes of one health subject categorizations were, namely, zoonoses, food safety, agriculture, environmental health and global health. The third objective analyzed the geographic distribution of scholarly one health resources, whether they were in developed nations or developing nations listed by the International Monetary Fund (IMF). The final objective reviewed trends in the application of the one health concept.

## 2. Materials and Methods

A cross-sectional study using Internet resources was carried out to analyze one health applications to zoonoses in scholarly resources from 1984 to 2012, representing a 28-year review. Before conducting the Internet search, clear definitions were made of the one health resources. Scholarly material was distinguished as eligible and ineligible using the following criteria which were found on Google Scholar and Ebscohost.

1. Peer-reviewed publications were classified as scientific journals and literature reviews of the pertinent subject matter (human, animal, and environmental health) that had been published in peer-reviewed journals.
2. Professional presentations were represented by formal presentations made by organizations and other professionals on the subject matter of human, animal, and environmental health, presenting research material, policy developments, or promotional activities in support of one health.
3. Grants and funding allocations were characterized as proposals for funding research, policy development, and so forth in the collaborative subject matter of humans, animals, and the environment accessed from reviewing all professional publications available from the systematic search conducted.
4. WHO-related reporting included updates from the website that involved relevant health issues, specifically reflecting the one health approach.
5. Book and book chapters were qualified as books or selections involving the subject matter.

The target population included all published studies that addressed the one health philosophy and which met the inclusion criteria. The documentation review included resources found on the Internet through the search engines and databases identified, which fit into the criteria of a one health approach and which took place from the concept's adoption of January 1, 1984. Excluded from this study were studies that were not found on the Internet databases, those that did not involve the one health concept, or fit the criteria of a one health approach, or those that were reported outside the period of study.

Database searches were conducted from May to July 2011. In the search fields for Google Scholar and Ebscohost, the terms “one health,” “health,” “human,” “animal,” and “zoonoses” were typed in. The first result that appeared from the database was reviewed and then assessed, using the definitions, to determine whether it fits into one of the one health approach criteria. Every fifth search result was examined and after reaching results on both databases numbered 130 onward, every second result was then considered. For every result that did not meet the inclusion criteria, the very next result was examined, and so forth, until a result did meet the criteria. After a result met the criteria, the fifth result from the last selected result was examined to be included in the review.

Each scholarly initiative that met the inclusion criteria was separated into its initiative category as well as into its year of publication. In addition, each resulting initiative was further categorized by the subject matter covered in the scholarly work. Considering one health scopes, these were the common subject areas covered: zoonoses, agriculture, food safety, environmental health, and global health. These categories were condensed from a larger, more complex list provided by the One Health Initiative Task Force [3]. For resources that contributed to more than one scope, such as agriculture and food safety, the final determination was made on the emphasis of one of the scopes from within the contents of the title. Finally, each initiative was also categorized into being conducted in, or having an analysis on, either a developed or developing nation based on a country's gross domestic product (GDP).

All the results were then categorized by their year of publication, the initiative that was represented, scope covered within the work, and the geographic distribution of where the initiative was conducted or what area was analyzed. SPSS statistical software package version 18.0 was used to analyze the frequencies of the years of scholarly resources, the initiative types, scopes categories, and geographic distribution.

## 3. Results

**Figure 1:**
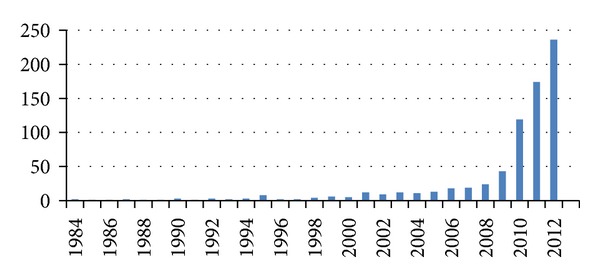
Frequency of recorded publications on one health between 1984 and 2012.

**Figure 2:**
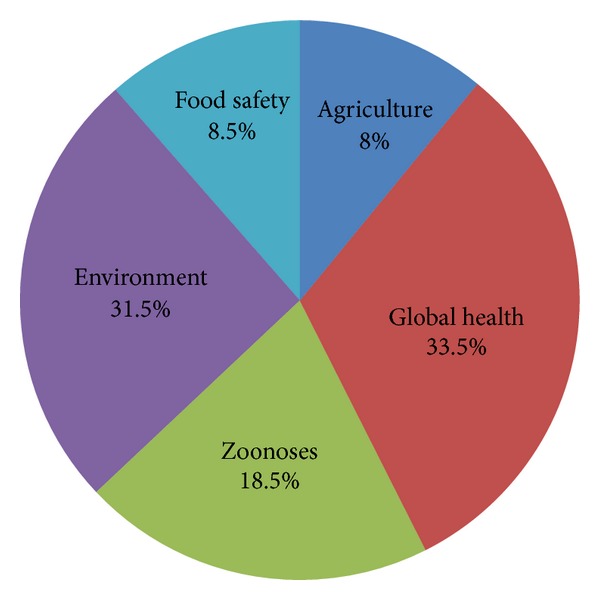
Distribution of reviewed published one health scopes.

**Figure 3:**
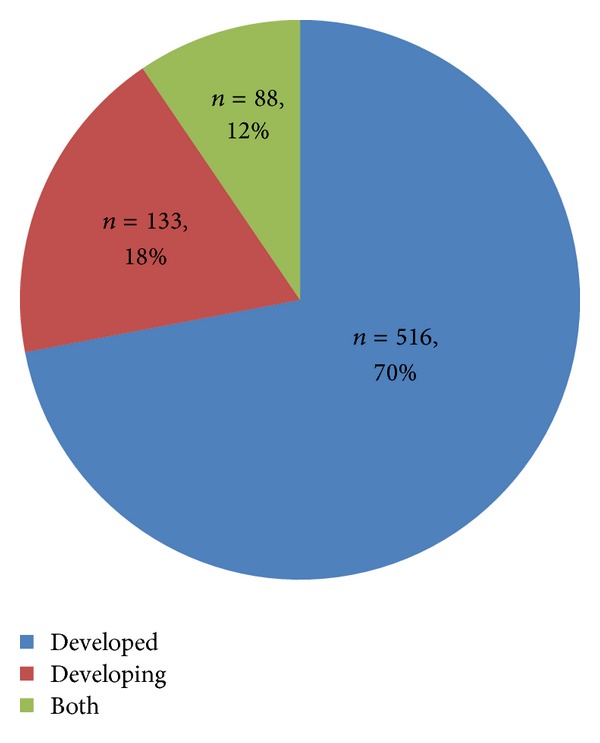
Distribution of the one health initiatives in developed versus developing countries.

All years for the review were represented, except for 1985, 1990, and 1991 as there were no publications that were sourced for these three years. There were a total of 8 resources in 1995 (4%) and 43 in 2009 (21.5%). The year 1998 began a continuous presence of one health resources annually. The year 2006 began a continued increase in one health resources for the period of review. The years 2010 to 2012 were the most productive for publications on one health as 71% of publications occurred during this period of time. An overall increase in the number of published one health scholarly works was found for the review with a marked increase in the most recent years (Figure 1). Journal articles, presentations, WHO reports, and books or book chapters were included in the analysis. Grants and funding allocations were not represented in the data gathering process. Of the resulting resources, peer-reviewed journal articles took precedence (85%) of all publications, while presentations and books accounted for 8.0% and 6.5%, respectively; only one WHO report was recorded. Evaluation of scopes, covered in the 737 scholarly resources (Figure 2), revealed that the predominant topics were global health, with 247 scopes (33.5%), and environmental health, with 232 total scopes (31.5%). In terms of geographic distribution of the scholarly resources, most of the resources focused their objectives within or towards countries that were already developed (70%) (Figure 3). An assessment on how one health initiatives were distributed by country size and GDP was achieved by mapping and measuring the burden of zoonoses and its distribution across the world (Table 1). Events of zoonoses were found to be disproportionately distributed as a result of the poverty and emerging market interface. Outbreaks or epidemics of emerging zoonoses were also noted to be sporadic in temporal and spatial distribution and appeared in developed countries where emerging zoonoses had not previously been reported but are increasing in incidence or geographical range.

Data on zoonoses extracted from the global burden of diseases noted that endemic zoonoses were concentrated among the developing countries of India, Nigeria, Democratic Republic of Congo, China, Ethiopia, and Bangladesh, whereas emerging zoonoses events were reported in the developed countries of the United States, United Kingdom, Australia, France, Brazil, Canada, Germany, and Japan (Table 1) [8].

For data analysis, chi-square was conducted to determine if, in the resulting reviewed years, one health resources themselves, scopes, and country locale differed significantly from the averages expected. Analysis revealed *P* values of less than 0.05 (*P* < 0.05), meaning that the resources, scopes, and country locale were all statistically different (Table 2).

Further analysis employed linear regression, using each focus, year, one health resource, scope, and country as the dependent variables and comparing them against independent variables of themselves. This showed whether the relationship between the independent and dependent variables was predictive or dependent on one another [9]. In the case of using year as the dependent variable, the regression shows that it was dependent on the initiative (*P* = 0.021), scope (*P* = 0.003) and the country locale (*P* = 0.021) (Table 3). Since all the values were <0.05, the null hypothesis was rejected and it was concluded that the years selected for the study showed a dependent relationship on the one health approach conducted, the scope topic areas and the represented country in the scholarly work.

The same linear regression was performed, this time using the initiative as a dependent variable against the other variables (Table 4). For this analysis, the initiative showed it to be dependent on the year (*P* = 0.022), as also noted in Table 3, but not dependant on scope (*P* = 0.643) nor on the country's locale (*P* = 0.465). The null hypothesis failed to be rejected because no complete dependency relationships were formed between all the variables from the regression test that was conducted, as compared to the regression testing done with year.

Next, scope was selected as the dependent variable against the year, initiative, and country. The linear regression showed that the scope was dependent on the year, as seen before (*P* = 0.003), but not dependent on the initiative (*P* = 0.643) nor on the country (*P* = 0.481) (Table 5). The null hypothesis thus failed to be rejected.

The country locale was used as the dependent variable against the others in the last linear regression. It was demonstrated that the country, whether developed or developing, was dependent on the year (*P* = 0.021) but not on the initiative (*P* = 0.445) nor the scope (*P* = 0.481) (Table 6). Again, the null hypothesis failed to be rejected for the whole dependency of scope on all other variables. The only rejected null was the dependency displayed between the year of the initiative and the initiative itself, its scope, and the country covered from within the initiative.

## 4. Discussion

Many of the results of this study could be attributed to the occurrences in the world during the time period of the study. When observing the trend of the one health approach over time, there was a minimal spike in 1995, an increased output from 2006, and marked increase from 2010 to 2012. Four (50%) of the eight scopes in 1995 were focused on environmental health, three involved global health (37.5%), and one scope involved food safety (12.5%). An environmental act was passed in 1995 in England by the Environment Agency and the Scottish Environment Protection Agency, Office of Public Sector Information [10]. It is likely that the passage of this law contributed to a higher production of publications. The increase in resources produced since 2006 could be related to the *E. coli* contamination incidents in the United States in 2006 and to the H1N1 outbreaks [11, 12] and also to the passing of the One Health Initiative Task Force in 2007 [3]. Two (4.7%) of the 43 defined resources in 2009 involved agriculture, eight involved environmental health (18.6%), 6 were on zoonoses (13.9%), 23 were on global health (53.5%), and four were on food safety (9.3%). The marked increase since 2010 may have resulted from the developments since 2006 which continued into 2009 which allowed for the one health approach to be placed on the research and scholarly agenda. 12 (5%) of the 236 recorded resources in 2012 involved agriculture, 50 (21%) involved environmental health, 56 (23.5%) were on zoonoses, 98 (41.5%) were on global health, and 21 (9%) were on food safety.

The distribution of the years of the one health approach, the scholarly resources, the scopes, and the countries' locale were not equally represented. For the one health concept to be appropriately beneficial to the global population, it would be necessary for a significant equal distribution of scholarly works to exist. The data, suggesting that the scopes of global health (33.5%) and environmental health (31.5%) dominated the others, including the zoonoses, produces an area of concern. The issues relating to one health, while in their genesis involved zoonoses and food safety, were identified as environmental and global health issues in the reporting and publications. While this shows evidence of the profound efforts to boost environmental and global knowledge about one health, it also demonstrated the limited body of knowledge of zoonoses, agriculture, and food safety.

Zoonoses, agriculture, and food safety are all interconnected topics in that they all directly impact the health of humans. In the last 30 years, there has been an average of one newly discovered emerging infectious disease every year [4]. A total of 335 emerging infectious diseases were identified between 1940 and 2004 [13]. Considering that more than 60% of infectious diseases are zoonotic, they have an important and increasing impact on human health. Agriculture, livestock production, and food safety practices are intimately linked with the prevention and control of zoonoses through the one health approach [14]. Considering the significance of agriculture and food safety, it was surprising that these scopes did not have a greater representation in the literature reviewed.

Developed countries, by virtue of their greater institutional facilities, trained personnel and financial resources are able to address the issues of one health approach. This is extremely beneficial as it enables developed nations to gain an awareness of one health initiatives and the added synergistic value of this approach. The One Health Initiative Task Force [3] has reported that while the developed countries prevail in making one health discoveries, it is the developing countries that suffer the most from the effects of zoonoses. It has been estimated that 70% of the reasons for poverty in Africa can be attributed to poor livestock production practices [15]. Zoonotic infections significantly impact animal production in this region further jeopardizing human and animal livelihoods.

The dependency of the initiative year, initiative, scope, and country locale on one another revealed that the incidence of the scope or country location is somewhat dependent on the year. In other words, it can be argued that the scope or country locale was represented due to that particular year, namely, due to the associated events during that year. Immediate action and scholarly resources are commonly implemented after a devastating event occurs [16], proving that the publishing of a particular one health topic may not be due to chance during that specific year. It is important to note that the general availability of one health resources is likely to be higher in the more recent years than in the 1980s, as the Internet was still in its evolutionary stage and not yet a global resource, as it is today [17]. The free availability of scholarly information on the Internet is evolving rapidly which will equalize the field. It will then be a matter of trained personnel and resources to make appropriate advances.

Many of the classifications which determined the scope of an initiative were subjective. Even though many of them clearly fit into their appropriate scope, some were hard to decipher, as some titles could have easily been included in more than one scope. As a result, one author's classification of an initiative could differ from another's opinion, resulting in interobserver bias. Some resources truly belonged in their own category; however, for the purposes of this study, only five scopes were included. This resulted in many resources being placed in the global health scope, as it is a category that could be applied to all one health approaches. Subjectivity was also a limitation in classifying the country locale. In some cases, resources' locations were clear from the article's title or content, and others were not. Some scholarly resources covered subject matter concerning a developing country, yet the actual work was conducted in a developed country.

The one health approach, according to the One Health Initiative, has been utilized to accelerate biomedical research discoveries, enhance public health efficacy, expeditiously expand the scientific knowledge base, and improve medical education and clinical care [18]. The increasing encroachment of people and livestock into wildlife habitats provided a multifaceted need to study bats and offer understanding for study at the human-wildlife interface [19]. Bats are an important reservoir and vector for spread of a number of emerging infectious diseases and they are associated with zoonoses with global public health significance such as Lyssa, Hendra and Nipah viruses, severe acute respiratory syndrome (SARS) like coronaviruses, and Ebola and Marburg viruses. The importance of wildlife as reservoirs of human diseases has also been widely recognized for most of the parasitic zoonoses, including American and African trypanosomiasis, leishmaniasis, giardiasis, cryptosporidiosis, balantidiasis, fascioliasis, opisthorchiasis, clonorchiasis, paragonimiasis, schistosomiasis, echinococcosis, taeniasis, diphyllobothriasis, sparganosis, dipylidiasis, trichinellosis, toxocariasis, strongyloidiasis, and *Ancylostoma caninum* and *A. braziliense* infections. Molecular phylogenetic methods used to examine the genetic diversity and species composition of these parasites in humans and their domestic and wild reservoir, paratenic, definitive, and intermediate host species have shown that they are in many instances identical. For example, African trypanosomes identified in wildlife in the Serengeti in Tanzania and the Luangwa Valley in Zambia which harbour a wide range of trypanosomes are the same species which infect humans and livestock [20]. The one health concept has successfully replaced the disease centered approach to zoonoses with a system based approach that aligns multiple disciplines, working locally, nationally, and globally, to attain optimal health for people, domestic, and wild animals and the environment.

Zoonotic diseases pose both major health threats and complex scientific and policy challenges, to which the social, cultural, and political norms and values are essential to address successful control outcomes [21]. The need to employ one health is illustrated in the cases of H5N1 avian influenza in which control failed due to the lack of addressing the complex dynamics of zoonotic diseases. Rapid Response Briefing [22] produced a report on the ebola haemorrhagic fever outbreak which occurred in Kibaale and Kampala in Uganda in 2012. The number of deaths in Kibaale was at least 16; the outbreak was spread 40 miles away to Kampala four months later. These two outbreaks demonstrated the continuing existence of ebola in Uganda which recorded an earlier outbreak in 2000 and led to 425 cases; more than half of the cases died.

The one health approach, employing disease surveillance, management, and eradication through collaboration between veterinarians dealing with livestock and wild animal populations and ecologists examining ecosystem biodiversity and public health experts, may have yielded a more rapid resolution to the outbreak The application of the one health approach has been recognized as a critical need by international organizations as well as the preferred approach to address global health issues. The 2013 Grand Challenges in Global Health [23] is based on the theme “The “One Health” Concept: Bringing Together Human and Animal Health for New Solutions.” The recent call for proposals for funding recognizes the lack of knowledge sharing and an artificial barrier that separates the fields of human and animal health. The Grand Challenges in Global Health specifically identified that advances in drug and vaccine discoveries for human diseases can be applied to provide tools and approaches for animal diseases that still plague developing countries. It is also noted that knowledge in veterinary medicine and animal nutrition and husbandry could provide insights into human nutrition and growth.

One health has gained momentum and now encompasses zoonotic infections, food safety, and even health delivery systems [24]. There is also an integrated epidemiological and economic framework for assessing zoonoses using a “one health” concept building on the medical focus of zoonoses [25]. In recent times the one health concept has been expanded to encompass the health and sustainability of the world's ecosystems [26]. Based on complex ecological thinking that goes beyond humans and animals, these approaches consider inextricable linkages beyond the human, animal, and environmental interface. Collaboration between veterinary, medical, and public health professionals to understand the ecological interactions and reactions to flux in a system can facilitate a clearer understanding of climate change impacts on environmental, animal, and human health. Climate change adds complexity and uncertainty to human health issues, such as emerging infectious diseases, food security, and national sustainability planning [27]. These issues intensify the importance of interdisciplinary and collaborative research.

Evidence for expanded application of one health compared to separate sectoral thinking is growing [28] and this integrative thinking is increasingly being considered in academic curricula in schools of medicine, veterinary medicine and public health [26], clinical practice, ministries of health and livestock/agriculture, and international organizations [29]. The one health approach to zoonoses however remains an average priority for health care professionals. The impact of zoonoses on animal health has been largely neglected but the effects on public health usually drive control initiatives on zoonoses and are much better defined by the use of Disability Adjusted Life Years (DALYS) [30]. The first zoonoses prioritization exercise involving health professionals in North America who had a limited knowledge of infectious diseases identified zoonoses as an area of priority [31]. Another study reported that local public health agencies in North America were not prepared and potentially unaware of their responsibility to be the initiator of the work on zoonotic disease information intelligence [32]. The advancement of the one health approach has increased the discussion and reporting on the topic. There remains a lack of knowledge and application of the integrated approach to health care by the health care professionals. Reaching the goal of control, and elimination and/or ultimate eradication of zoonoses pose a significant challenge for the future. Standardized interlaboratory test validation, intersectoral collaboration and establishment of an international one health diagnostic platform are considered to be important strategies [33]. The sharing of best practices on diagnosis of zoonoses and the further refinement of new, cheaper, multispecies tests which can be interpreted by minimally trained individuals could contribute to a greater level of intersectoral integration, control, and elimination of zoonoses. The projection from one health may eventually lead to a one system approach based on the inherent challenges to intersect disciplines that belong to different systems. One health approaches applied across international boundaries that share the same challenges are required to create sustainable and coordinated control. The one system approach focusing on the strengthening of the community model health system as a whole as well as developing effective and novel tools to be applied across all aspects of health, is fundamental of a one world one health approach [34]. The future of one health is a one world approach with the continued effort towards integration of the contributing parts that form the whole which is health.

## 5. Conclusions

The one health approach continues to be a highly investigated concept, via the pursuit of scholarly resources involving the health of humans, animals, and the environment. There is a need to increase research on zoonoses, food safety, and agriculture and to improve the understanding of the one health concept. This could be achieved by introducing more scholarly resources in developing countries by the further development of the Internet and the free availability of online information on one health. The use of Massive Open Online Courses (MOOC) available to developing countries is now being offered to deliver courses on the approach and applications of one health [35]. This is critical because most of the public health and economic impacts that occur within the concept of one health occur in developing nations. The lack of basic health infrastructure in developing countries means that everything else suffers as a result, namely, the environment, human, and animal health and well-being. The future of one health is at a crossroad; there is a need to more clearly define its boundaries and demonstrate its benefits. The greatest acceptance of one health is seen where it is having significant impacts on control of infectious diseases. There is also a continuing need for further efforts towards integration with the global community serving as the unit of a one system approach.

## Figures and Tables

**Table 1:** Interface of zoonoses and the global burden of disease. Adapted from [8].

Poverty interface		Emerging market interface		Zoonoses interface		
Poor livestock keepers	Protein energy malnutrition	Monogastrics (TLU) 2010	Rapid change monogastrics 2010–2030	Zoonoses burden GBD	Endemic zoonoses prevalence	Emerging zoonoses events
India	India	China	Myanmar	India	Nigeria	USA
Nigeria	Ethiopia	Brazil	Burkina Faso	Nigeria	Ethiopia	UK
Ethiopia	Nigeria	Indonesia	India	Congo DR	Tanzania	Australia
Bangladesh	China	India	Pakistan	China	Togo	France
Congo DR	Congo DR	Vietnam	Ghana	Ethiopia	India	Brazil
Pakistan	Bangladesh	Iran	Afghanistan	Bangladesh	Mali	Canada
Kenya	Pakistan	Philippines	Bangladesh	Pakistan	Vietnam	Germany
Sudan	Indonesia	Thailand	Liberia	Afghanistan	Sudan	Japan
China	Angola	Nigeria	Central African Republic	Angola	Bangladesh	China
Tanzania	Afghanistan	Ukraine	Chad	Brazil	Burkina	Sweden
Indonesia	Tanzania	Pakistan	Cambodia	Indonesia	Cameroon	Italy
Madagascar	Brazil	Myanmar	Benin	Niger	Chad	Malaysia
Niger	Philippines	Bangladesh	Laos	Tanzania	Rwanda	Switzerland
Uganda	Uganda	Peru	Thailand	Kenya	Ghana	Congo DR
Turkey	Mali	Colombia	Zimbabwe	Côte d'Ivoire	Mozambique	Sudan
Philippines	Sudan	Ecuador	Ethiopia	Uganda	South Africa	Argentina
Afghanistan	Mozambique	Morocco	Guinea	Sudan	Congo DR	India
Egypt	Malawi	South Africa	Guinea-Bissau	Burkina	Egypt	Israel
Mozambique	South Africa	Bolivia	China	Mali	Gambia	Peru
Burkina	Vietnam	Egypt	Mali	Iraq	Ivory Coast	Trinidad and Tobago
					Pakistan	Uganda
					Zimbabwe	Vietnam

**Table 2:** Analysis of one health initiatives by year, scopes, and country.

	Year	Initiative	Scope	Country
Chi-square	253.100	386.520	31.600	79.380
Df	22	3	4	1
Asymp. sig.	0.000	0.000	0.000	0.000

**Table 3:** Analysis of the one health initiatives by year, scopes, and country. Dependent variable: year.

Model	Unstandardized coefficients		Standardized coefficients	*t*	Sig.
	*B*	Std. error	Beta		
1					
(Constant)	2000.749	1.374		1456.344	0.000
Initiative	−0.813	0.364	−0.153	−2.233	0.021
Scope	0.879	0.305	0.197	2.881	0.003
Country	2.145	0.956	0.154	2.244	0.021

**Table 4:** Analysis of the one health initiatives by year, scopes, and country. Dependent variable: initiative.

Model	Unstandardized coefficients		Standardized coefficients	*t*	Sig.
	*B*	Std. error	Beta		
1					
(Constant)	62.541	27.326		2.289	0.023
Year	−0.031	0.014	−0.162	−2.233	0.022
Scope	0.031	0.060	0.036	0.507	0.643
Country	−0.156	0.187	−0.060	−0.835	0.465

**Table 5:** Analysis of the one health initiatives by year, scopes, and country. Dependent variable: scope.

Model	Unstandardized coefficients		Standardized coefficients	*t*	Sig.
	*B*	Std. error	Beta		
1					
(Constant)	−89.561	32.125		−2.788	0.006
Year	0.046	0.016	0.207	2.881	0.003
Initiative	0.043	0.084	0.036	0.507	0.643
Country	−0.157	0.222	−0.050	−0.707	0.481

**Table 6:** Analysis of the one health initiatives by year, scope, and country. Dependent variable: country.

Model	Unstandardized coefficients		Standardized coefficients	*t*	Sig.
	*B*	Std. error	Beta		
1					
(Constant)	−22.504	10.421		−2.159	0.032
Year	0.012	0.005	0.163	2.244	0.021
Initiative	−0.023	0.027	−0.060	−0.835	0.445
Scope	−0.016	0.023	−0.051	−0.707	0.481
